# Force dysmetria in spinocerebellar ataxia 6 correlates with functional capacity

**DOI:** 10.3389/fnhum.2015.00184

**Published:** 2015-04-08

**Keywords:** SCA6, dysmetria, functional capacity, force control, goal-directed

## Abstract

Spinocerebellar ataxia type 6 (SCA6) is a genetic disease that causes pure cerebellar degeneration affecting walking, balance, and coordination. One of the main symptoms of SCA6 is dysmetria. The magnitude of dysmetria and its relation to functional capacity in SCA6 has not been studied. Our purpose was to quantify dysmetria and determine the relation between dysmetria and functional capacity in SCA6. Ten individuals diagnosed and genetically confirmed with SCA6 (63.7 ± 7.02 years) and nine age-matched healthy controls (65.9 ± 8.5 years) performed goal-directed isometric contractions with the ankle joint. Dysmetria was quantified as the force and time error during goal-directed contractions. SCA6 functional capacity was determined by ICARS and SARA clinical assessments. We found that SCA6 participants exhibited greater force dysmetria than healthy controls (*P* < 0.05), and reduced time dysmetria than healthy controls (*P* < 0.05). Only force dysmetria was significantly related to SCA6 functional capacity, as measured with ICARS kinetic score (*R^2^* = 0.63), ICARS total score (*R^2^* = 0.43), and SARA total score (*R^2^* = 0.46). Our findings demonstrate that SCA6 exhibit force dysmetria and that force dysmetria is associated to SCA6 functional capacity. Quantifying force and time dysmetria in individuals with SCA6 could provide a more objective evaluation of the functional capacity and disease state in SCA6.

## Introduction

Spinocerebellar ataxia type 6 (SCA6) is caused by a genetic mutation that results in pure5 cerebellar degeneration (Sasaki et al., 1998; Solodkin and Gomez, 2012). Common symptoms of SCA6 include impaired walking, balance, and movement coordination (Morton and Bastian, 2004). Another typical symptom of SCA6 is dysmetria, which refers to the inability to perform accurate movements (Solodkin and Gomez, 2012). The magnitude of dysmetria and its relation to functional capacity has not been well-studied in SCA6. The focus of this study was to compare single-joint dysmetria in individuals with SCA6 and healthy controls and examine the relation of single-joint dysmetria to the functional capacity of SCA6.

The isolated cerebellar degeneration in SCA6 typically has an adult-onset (45 ± 14 years; Solodkin and Gomez, 2012). Because the cerebellum is pivotal in the control of voluntary limb movements, eye movements, balance, and locomotion (Morton and Bastian, 2004), typical symptoms of individuals with SCA6 include gait ataxia (loss of balance and impaired locomotion), dysarthria, nystagmus, and dysmetria (Solodkin and Gomez, 2012). During clinical exams, dysmetria is assessed through the finger-to-nose test (Storey et al., 2004; Saute et al., 2012). Individuals are required to move their finger from their nose to a target quickly and accurately. Such voluntary movements in SCA6 are characterized by significant overshooting and undershooting of the targeted endpoint (Storey et al., 2004; Saute et al., 2012).

The literature on goal-directed contractions in older adults provides insight into understanding errors and dysmetric movements. Single-joint goal-directed contractions have been used as a model to understand age-related differences in force and time errors (Christou, 2011). Healthy older adults exhibit greater force and time errors compared with young adults, especially at low levels of force. This finding has been consistent across goal-directed contractions with different joints and has been shown during index finger abduction (Christou et al., 2007), elbow flexion (Chen et al., 2014), knee extension (Christou and Carlton, 2002), and ankle dorsiflexion (Chen et al., 2014). Although dysmetria can have functional consequences by limiting the accuracy of voluntary contractions, it remains unclear how it relates to the functional capacity of individuals with ataxia.

Individuals with SCA6 present an outstanding model to further understand the role of dysmetria to functional capacity. It is well-established that individuals with SCA6 exhibit functional impairments, which can be assessed reliably with clinical assessments (e.g., International Cooperative Ataxia Rating Scale (ICARS) and the Scale for the Assessment and Rating of Ataxia (SARA) (Schmitz-Hübsch et al., 2006b; Saute et al., 2012). The purpose of this study, therefore, is to quantify force and time dysmetria in SCA6 and examine the relation of dysmetria to the functional capacity in SCA6. To accomplish this, we examined dysmetria of a goal-directed ankle dorsiflexion task and quantified the functional capacity with ICARS and SARA clinical assessments. We hypothesized that SCA6 compared with healthy controls would exhibit greater dysmetria during the goal-directed task and that dysmetria will correlate to the functional capacity of SCA6.

## Materials and Methods

### Participants

Ten individuals diagnosed and genetically confirmed with SCA6 (63.7 ± 7.02 years; four males, six females) and nine age-matched healthy controls (65.9 ± 8.5 years; four males, five females) volunteered to participate in this study. Clinical characteristics of the SCA6 participants are shown in **Table 1**. Control participants reported being healthy and having no neurological impairments. The Institutional Review Board at the University of Florida approved the procedures of this study. All participants signed a written informed consent before participating in the study.

**Table 1:** Description of SCA6 participants.

ID	Age (years)	Sex	ICARS kinetic score	ICARS total score	SARA total score
SCA1	52	F	11	27	8.5
SCA2	73	M	7	15	5
SCA3	59	M	11	32	12
SCA4	57	F	19	55	21
SCA5	61	M	19	37	17
SCA6	75	F	7	23	12
SCA7	66	F	6	20	7
SCA8	62	F	6	20	9.5
SCA9	66	M	5	13	4.5
SCA10	66	F	13	28	13

### Experimental Protocol

There were two sessions for SCA6 participants and one session for healthy controls. The first session for SCA6 participants was the evaluation through clinical assessment with a neurologist and lasted ∼45 min. The second session for SCA6 and the single session for healthy controls involved performing goal-directed ankle dorsiflexion contractions and lasted 1 h. In this session, we explained the experimental procedures and the goal-directed single-joint contraction task. Following the explanation of the task participants performed the following: (1) maximum voluntary contraction (MVC) task with ankle dorsiflexion; (2) practice of 3–5 goal-directed contraction trials at a different target from the actual target; (3) 50 goal-directed contraction trials.

### Experimental Procedures

#### Clinical Assessments

Functional capacity of SCA6 participants was evaluated through two different clinical assessments. Participants visited a neurologist who performed the ICARS (Schmitz-Hübsch et al., 2006b) and the SARA (Schmitz-Hübsch et al., 2006a) in one session of ∼45 min. These two assessments are the most commonly used clinical rating scales to evaluate disease severity and progression in people with SCA (Saute et al., 2012). Control participants were not evaluated with ICARS and SARA.

The ICARS is a 100 point clinical rating scale. It includes 19 items divided into four sub-scales: posture and gait disturbance (seven items, 34 points); kinetic function (seven items, 52 points); speech disorder (two items, eight points); and oculomotor disorder (three items, six points; Storey et al., 2004; Saute et al., 2012). The ICARS Kinetic score is the score obtained in the kinetic function sub-scale. This sub-scale evaluates motor control of the limbs by looking for limb coordination, intention tremor, and dysmetria (Saute et al., 2012). The SARA is a 40 point rating scale. SARA has only eight items which include gait (eight points), stance (six points), sitting (four points), speech disturbance (six points), finger chase (four points), nose-to-finger test (four points), fast alternating hand movements (four points), and heel-sheen slide (four points; Schmitz-Hübsch et al., 2006a; Yabe et al., 2008). In these two clinical assessments a higher score is indicative of more severe ataxia.

#### Experimental Setup and Apparatus for Goal-Directed Contractions

Each participant was seated comfortably in an upright position and faced a 32 inch monitor (Sync Master^TM^ 320MP-2, Samsung Electronics America, Ridgefield Park, NJ, USA) that was located 1.25 m away at eye level. The monitor was used to display the contraction produced by ankle dorsiflexion using a custom-written program in Matlab (Math Works^TM^ Inc., Natick, MA, USA). All participants affirmed that they could see the display clearly.

**FIGURE 1:**
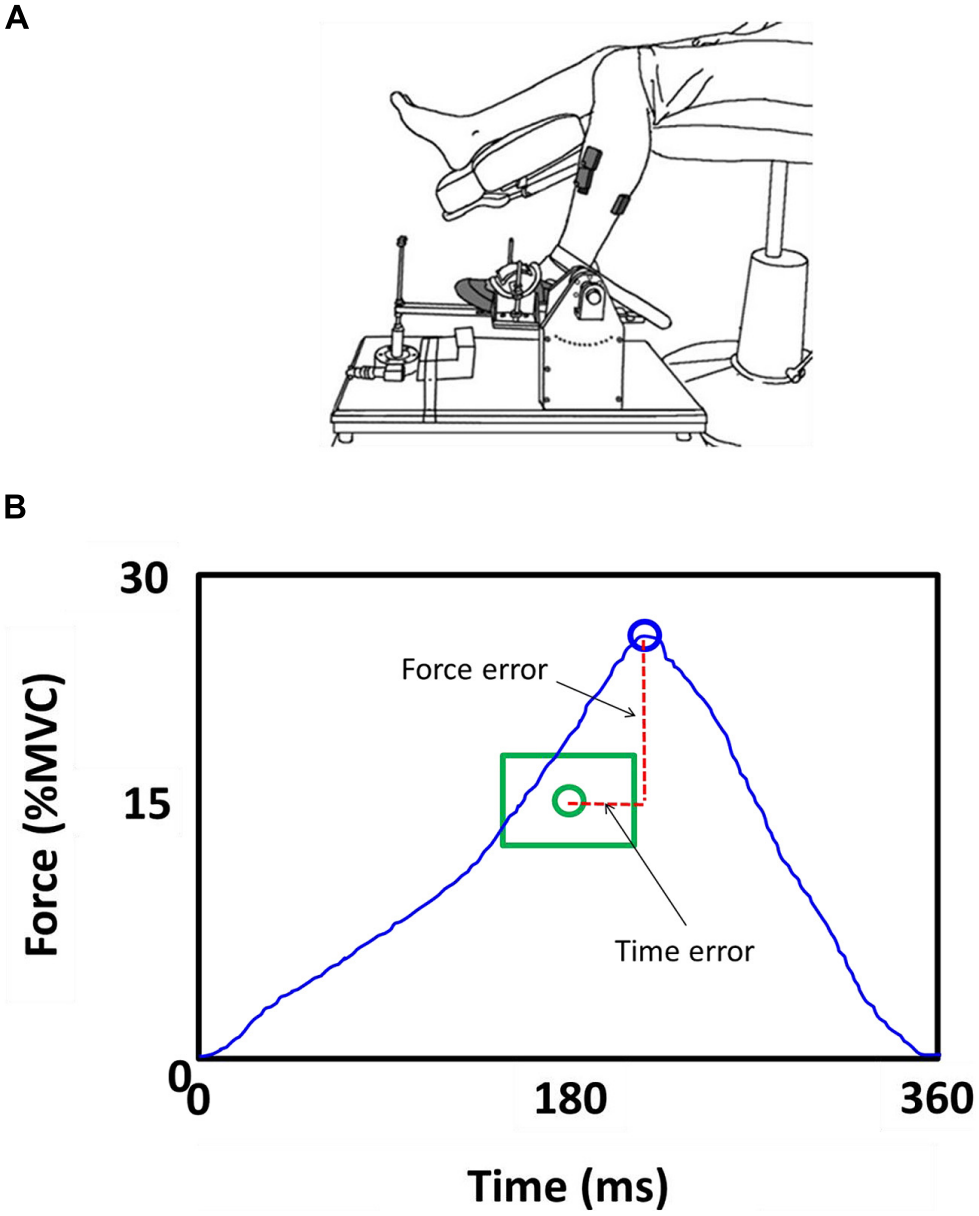
Goal-directed task. **(A)** Subjects were seated with the hip, knee, and ankle joint at 90°. The left foot was restrained in an ankle device. Subjects performed a goal-directed ankle dorsiflexion contraction with the left foot. **(B)** Force and time-to peak force errors were normalized to the targeted force and time.

For the isometric goal-directed task, the left hip joint was flexed to ∼90° and abducted by ∼10°, and the knee was flexed to ∼90°. The left foot rested on a customized foot device with an adjustable foot plate and was secured by straps over the metatarsals to ensure an isolated dorsiflexion of the ankle (**Figure 1A**). The initial ankle position was ∼90° of ankle dorsiflexion. We chose the left leg for this study because it was the non-dominant limb and thus the task would be more difficult for the participants compared with the dominant limb (Christou et al., 2007).

#### Force

The maximum voluntary force exerted during MVC ankle dorsiflexion measurements and the force exerted during the goal-directed contraction were measured using a force transducer (Model MB-100, Interface, Scottsdale, AZ, USA) that was located in parallel with the force direction on the customized foot device. The ankle force signal was sampled at 1000 Hz with a NI-DAQ card (Model USB6210, National Instruments, Austin, TX, USA), and stored on a personal computer.

#### MVC Task

We identified the MVC for ankle dorsiflexion before the 50 trials of the goal-directed task. Participants increased force to their maximum in 3 s and maintained the maximal force for 3 s. They exerted 3–5 MVCs or until two MVC trials were within 5% of each other. One-minute rest was provided between consecutive trials to minimize fatigue.

#### Goal-Directed Contractions

Participants performed isometric ankle dorsiflexion contractions that require them to accurately match a force and time to target. The targeted force was 15% of their MVC and the targeted time to peak force was 180 ms.

The task was divided in three phases: (1) GET READY; (2) CONTRACT; and (3) FEEDBACK. The GET READY phase began by the presence of a red target on the monitor for 2 s. This was a cue for the participants to be ready for the CONTRACT phase. The CONTRACT phase began when the red target changed its color to green. This change in color was the cue for participants to perform the goal-directed contraction. There was no feedback of the force exerted by the participant during this phase to eliminate any possibility of force and time adjustments during the contraction. The green target stayed on the monitor for 3 s and participants were instructed to perform the contraction at their convenience (not a reaction time task). The recording of the task began when the participant initiated the contraction within the 3 s of the CONTRACT phase. The FEEDBACK phase began at the end of each CONTRACT phase and lasted for 5 s. We provided the participants with visual feedback of their contraction trace relative to the targeted force-time endpoint (**Figure 1B**). This feedback allowed the participants to better understand the task, adapt and improve (short-term learning) their performance. A graphical representation of this part of the task has been shown previously (Chen et al., 2014). The visual gain was kept constant at 1° (visual angle) for all trials (Vaillancourt et al., 2006; Christou, 2011).

### Data Analysis

Data was analyzed off-line using custom-written programs in Matlab (Math Works^TM^ Inc., Natick, MA, USA). We calculated the force and time endpoint accuracy during the ankle dorsiflexion goal-directed task. We eliminated a very small number of trials (less than 5% of the total trials for both groups) based on the criteria that the force and time errors were ±2 SD of the median performance.

#### MVC Force

The highest force exerted with ankle dorsiflexion, during the MVC task.

#### Force and Time Dysmetria

To calculate force and time dysmetria we quantified the force and time errors. Force error was quantified as the absolute deviation from the targeted peak force (force dysmetria), whereas time error was quantified as the absolute deviation from the targeted time to peak force (time dysmetria; ms). The force error was normalized to the targeted peak force (Eq. 1).

$$Force\,\;\,error\,\;\left(\%MVC\right)=(15*\frac{peak\,\;\;force\left(N\right)}{\;t\,\arg eted\,\;\;\;peak\,\;\,force})\;\;\;\;\;\left(1\right)$$

### Statistical Analysis

The major dependent variables were: (1) force error (%MVC); (2) time error (ms); (3) ICARS kinetic score; (4) total ICARS score; (5) total SARA score. We compared force and time error for SCA6 and healthy controls using an independent *t*-test. To determine the relation between dysmetria and functional capacity we used a linear regression analysis. The goodness-of-fit of each regression was given by the squared correlation (*R^2^*; Green and Salkind, 2010). All statistical analyses were performed with the IBM statistics 21.0 statistical packages (IBM Inc., New York). The alpha level for all statistical tests was 0.05. Data are reported as mean ± SD within the text and as mean ± standard error of the mean (SEM) in the figures.

## Results

### MVC Force

The MVC force for ankle dorsiflexion was not significantly different between healthy controls and SCA6 (*P* = 0.6). Healthy controls exerted 113.0 ± 55.7 N of MVC force with the ankle dorsiflexors, whereas the SCA6 exerted 127.3 ± 64.4 N of MVC force with the ankle dorsiflexors.

### Dysmetria

**FIGURE 2:**
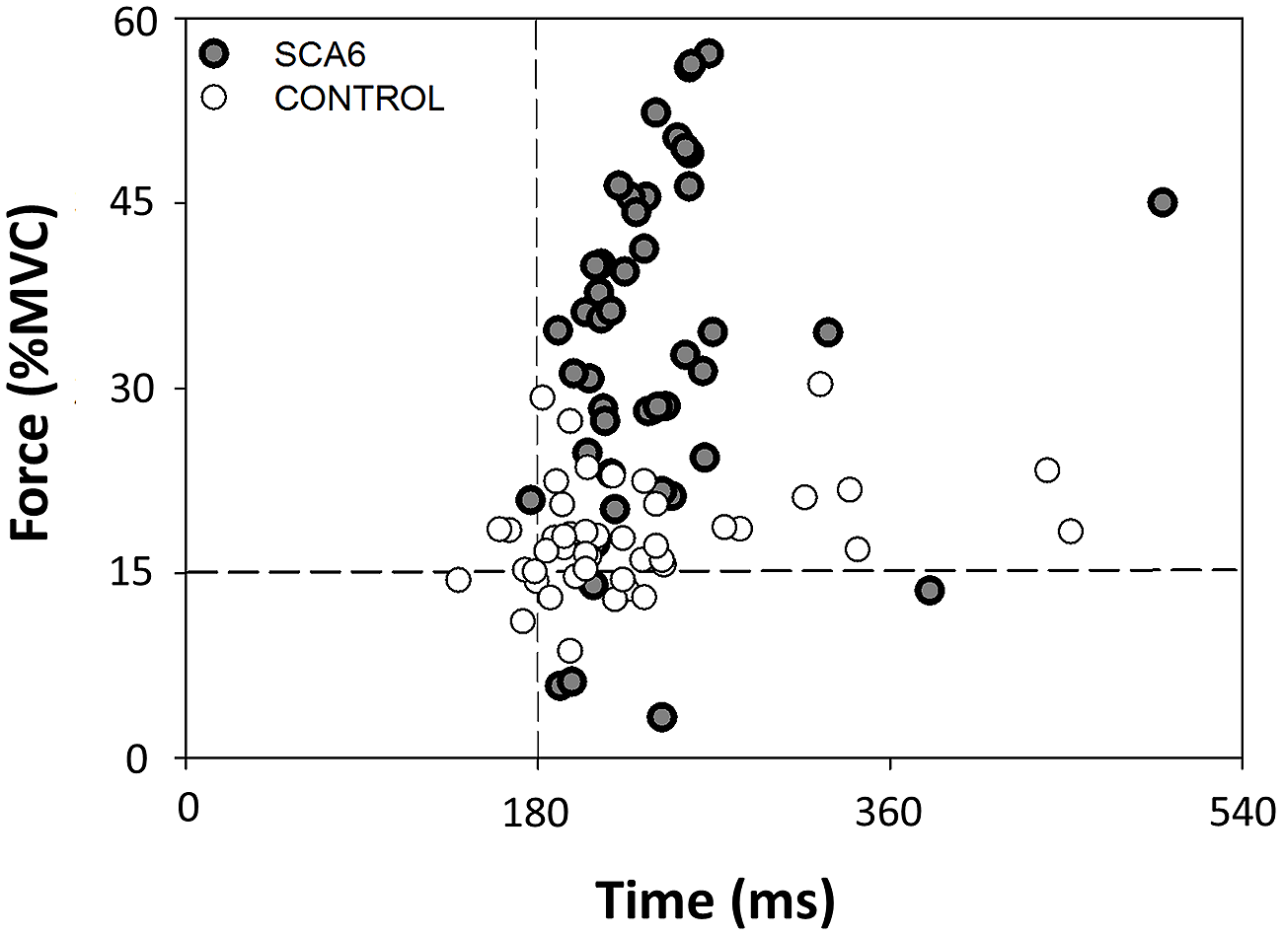
Representative end-points of the 50 trials during the goal-directed task for a SCA6 and an age-matched control participant relative to the target. The SCA6 participant exhibited greater force dysmetria than the control participant.

**FIGURE 3:**
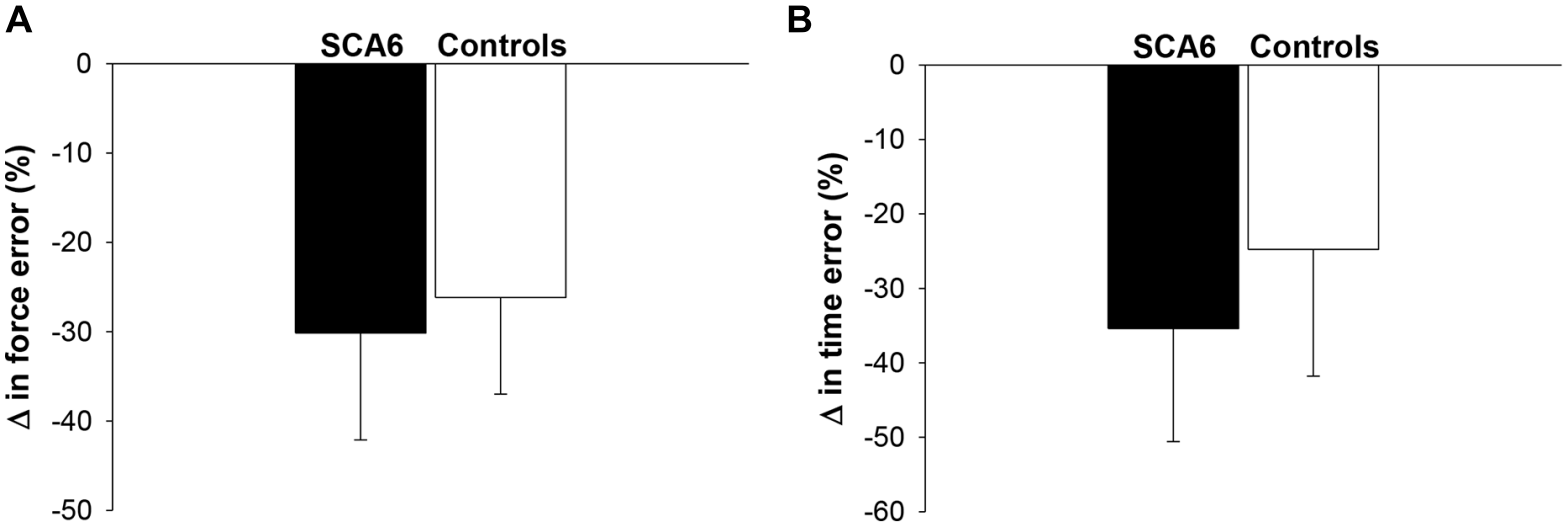
Changes in force and time error during task acquisition. The change in force error **(A)** and time error **(B)** from the first block to the last block of practice was similar for SCA6 participants and healthy controls.

**FIGURE 4:**
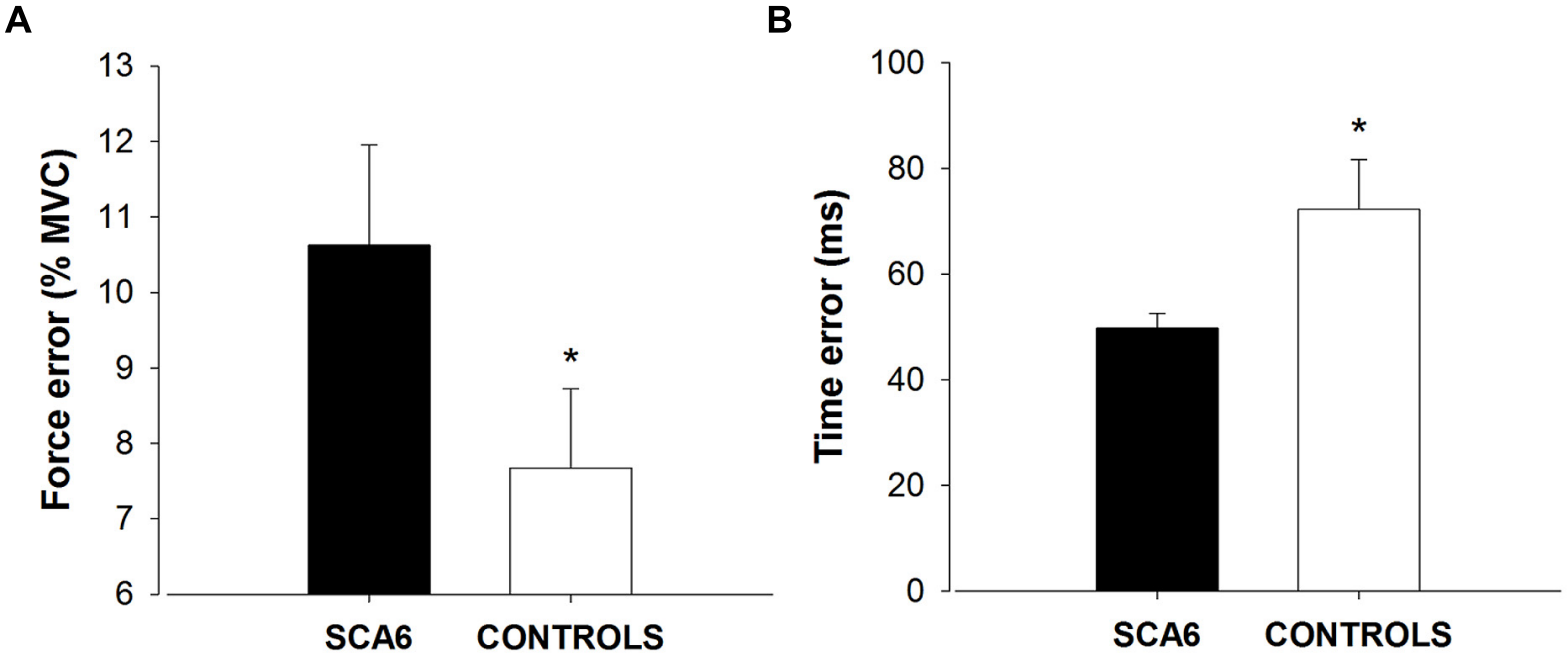
Force and time dysmetria. SCA6 participants exhibited significantly greater force error (force dysmetria; **A**) and lower time error **(B)** compared with healthy controls. * Indicates significant difference (*P* < 0.05) in force and time error between SCA6 and controls.

We quantified force and time dysmetria with the force and time endpoint accuracy of an ankle dorsiflexion goal-directed task. **Figure 2** provides a representative result from one SCA6 participant and one healthy control participant. Task acquisition was similar for SCA6 and controls, as it is evident from similar changes in force (**Figure 3A**; *P* > 0.2) and time error (**Figure 3B**; *P* > 0.2). Overall, SCA6 exhibited greater ankle dorsiflexion force error (**Figure 4A**; *t* = 1.74, *P* < 0.05) and lower time error (**Figure 4A**; *t* = -2.27, *P* < 0.05) than healthy controls.

### Dysmetria and Clinical Assessment Scores

**FIGURE 5:**
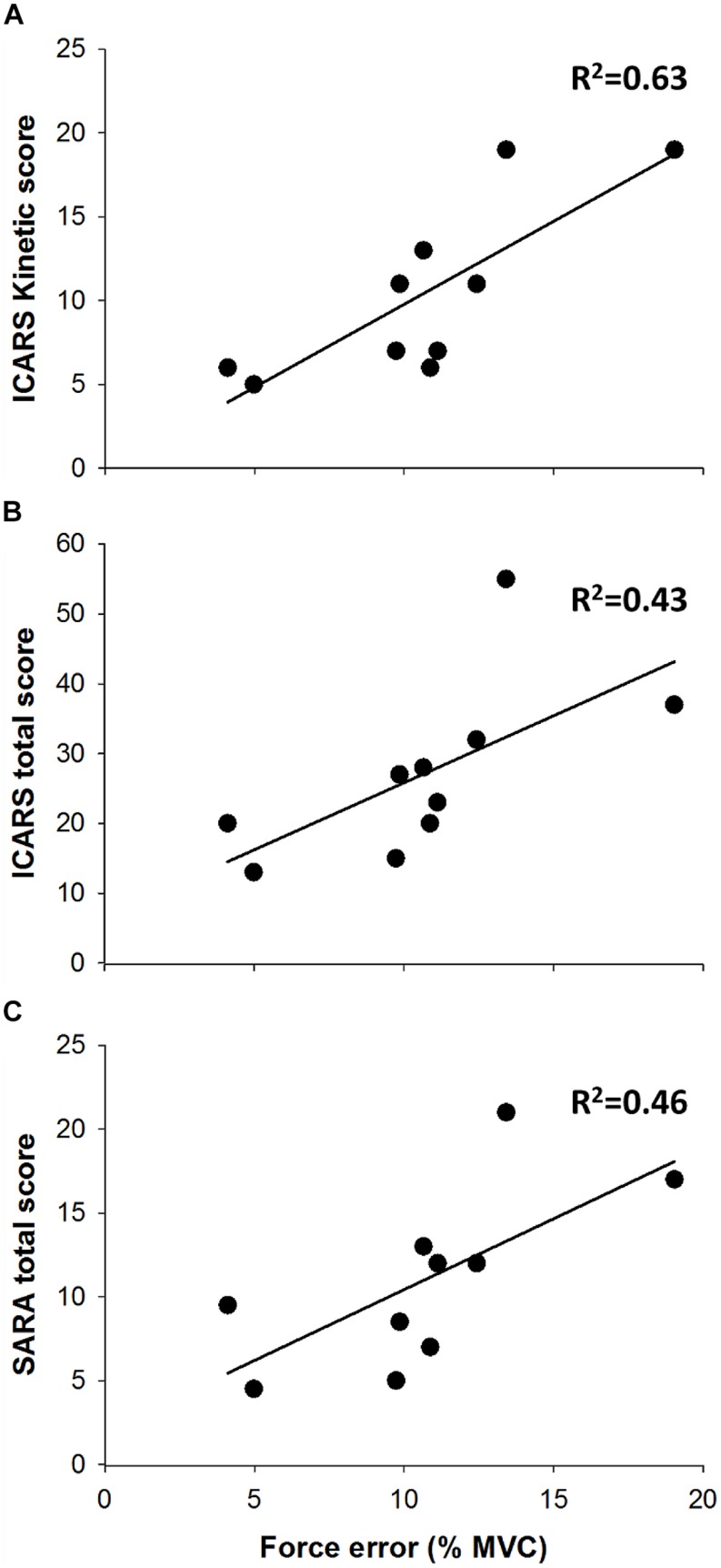
Force dysmetria and clinical assessment. Force dysmetria was strongly associated with ICARS kinetic score (**A**; *R^2^* = 0.63), and moderately associated with ICARS Total Score (**B**; *R^2^* = 0.46) and SARA Total Score (**C**; *R^2^* = 0.46).

The above results suggest that SCA6 exhibit greater force dysmetria but lower time dysmetria than healthy controls. To determine the contribution of force and time dysmetria to the functional capacity of individuals with SCA6, we correlated the force and time errors with the clinical assessment scores. Force dysmetria was positively related with the ICARS kinetic score (*R^2^* = 0.63, *P* = 0.006; **Figure 5A**), the ICARS Total score (*R^2^* = 0.43, *P* = 0.04; **Figure 5B**), and SARA Total score (*R^2^* = 0.46, *P* = 0.03; **Figure 5C**). In contrast, time dysmetria was not significantly related with the ICARS kinetic score (*R^2^* = 0.15, *P* > 0.2), the ICARS Total score (*R^2^* = 0.12, *P* > 0.2), and SARA Total score (*R^2^* = 0.2, *P* > 0.2).

## Discussion

In this study, we aimed to quantify force and time dysmetria in SCA6 and examine the relation between dysmetria and the functional capacity of SCA6. As expected, our results suggest that force dysmetria is significantly greater in SCA6 than healthy controls. In contrast to our expectation, SCA6 exhibited lower time dysmetria than healthy controls. Nonetheless, only force dysmetria correlated to the clinical assessment scores in SCA6. Therefore, these findings provide novel evidence that only force endpoint control is impaired in SCA6 and relates to their functional capacity. Dysmetria during goal-directed contractions, therefore, may be a useful tool to evaluate disease state in SCA6.

### Dysmetria in SCA6

Dysmetria is characterized by the overshoot or undershoot of targeted endpoints during voluntary movements. Although dysmetria is one of the main symptoms of individuals with SCA6, the magnitude of dysmetria in SCA6 has not been quantified. In this study we quantified dysmetria through errors during goal-directed contractions. We found that force dysmetria is exacerbated in SCA6 participants compared with healthy controls and force dysmetria correlates with functional capacity in SCA6.

Increased force dysmetria in SCA6 may be related to at least two factors: (1) Cerebellar degeneration may influence the input to the thalamus and consequently to the motor cortex. This deficiency in the cerebellar-thalamo-cortical loop may increase force variability and consequently increase force dysmetria (Christou et al., 2007). (2) Cerebellar degeneration may limit acquisition and generation of accurate inverse models (Bays and Wolpert, 2007). The cerebellum is acting as the comparator between the efference copy and afferent feedback. Given that afferent feedback is intact in SCA6 (Morton and Bastian, 2004), it is possible that the deficiency occurs in the ability of the cerebellum to compare the efference copy and afferent feedback and consequently form an inaccurate motor plan. Future studies should distinguish the contribution of these two factors to dysmetria in SCA6.

Interestingly, recent evidence from Bastian’s group suggests that cerebellar degeneration impairs the control of muscle forces of the arm (Charles et al., 2013). Individuals with cerebellar ataxia had difficulty performing accurate isometric contractions to match the force magnitude and direction of a goal-directed task with their arm (Charles et al., 2013). This finding is important because it demonstrates that the mechanism of dysmetria with cerebellar degeneration may be independent of impairments in the ability to compute and coordinate limb dynamics. Our finding, therefore, expands the literature demonstrating that SCA6 exhibit greater force dysmetria than healthy controls to the lower limb.

Furthermore, our findings may hold implications for considering rehabilitation protocols to improve force dysmetria in individuals with SCA6. Indeed, prior training studies of patients with cerebellar damage have shown that improving dysmetria via coordination training appears to be beneficial to the walking ability of cerebellar patients (Ilg et al., 2009). It is possible that force dysmetria could be a potential factor during features of the ataxic gait and diminished functional capacity in SCA6. As a consequence, we examined the relation between force dysmetria and functional capacity in SCA6 participants.

### Dysmetria and Functional Capacity

Traditionally, functional capacity and disease progression in individuals with SCA6 has been quantified qualitatively through clinical assessments (Saute et al., 2012). In this study, we correlated ankle dysmetria and the clinical assessment scores in SCA6. For the first time, we examined whether single joint dysmetria is associated with impaired function in SCA6. We found that ankle force dysmetria is positively associated to clinical assessment scores. Greater force dysmetria (increased force endpoint error) in SCA6 was associated with lower functional capacity (higher clinical assessment scores). This result suggests that dysmetria could be an underlying mechanism of ataxic gait and functional capacity of SCA6.

To our knowledge this is the first paper that provides evidence that dysmetria correlates to the functional capacity in SCA6. However, it is known that greater dysmetria impairs motor performance in older adults. For example, greater force error results in impaired manual force control in older adults (Christou et al., 2007). Furthermore, training that reduces force errors results in improved manual dexterity in older adults (Kornatz et al., 2005). Our findings provide novel evidence that increased dysmetria in SCA6 is associated with reduced functional capacity and could aid the evaluation of disease state in SCA6. Further studies that examine progression of SCA6 should consider evaluating force dysmetria.

### Limitations and Future Considerations

In this study the goal-directed task consisted of one predetermined force and time target. Although our results indicate that SCA6 participants have intact time control, it could be possible that the single time to target limits our ability to understand time dysmetria in SCA6. Future studies should examine SCA6 performance through a variety of targeted times and force intensities to better understand dysmetria in SCA6.

Moreover, this study only examines the ankle joint during isometric dorsiflexion. Future studies could focus on dysmetria during movements to understand the dynamic components of dysmetria and its relation to functional capacity. Finally, evaluating dysmetria in different joints (e.g., elbow flexion and reaching movements) is needed for an overall understanding of dysmetria in SCA6.

## Summary

The observed relation between force dysmetria and functional capacity in SCA6 suggests that dysmetria could be a potential tool to evaluate individuals with SCA6. Using dysmetria as an indicator of functional capacity could lead to more objective assessments of disease progression in SCA6. Force dysmetria during a goal-directed task could allow the detection of subtle changes in the disease that cannot be detected by the global observations of clinical assessments. Therefore, quantifying force dysmetria with a goal-directed task provides an objective way of measuring functional capacity in SCA6.
